# The psychosocial implications of cervical cancer in women living in sub-Saharan Africa

**DOI:** 10.4102/curationis.v48i1.2618

**Published:** 2025-02-18

**Authors:** Johanna E. Maree, Nokuthula G. Nkosi, Agnes A. Huiskamp

**Affiliations:** 1 Department of Nursing Education, Faculty of Health Sciences, University of the Witwatersrand, Johannesburg, South Africa

**Keywords:** cervical cancer, psychosocial implications, sub-Saharan Africa, nursing, scoping review

## Abstract

**Background:**

A cervical cancer diagnosis has several implications for women’s lives. Living with cervical cancer in the context of sub-Sahara Africa’s unique challenges can have a devastating effect on psychosocial health.

**Objectives:**

This study describes the publication output reporting psychosocial implications of cervical cancer for women living in sub-Saharan Africa.

**Method:**

A scoping review was conducted using the keywords Africa and cervical cancer in combination with psychosocial, psychological, social, spiritual, cultural and financial to search five databases. A data extraction sheet was developed to capture the relative data, which was analysed using content analysis and descriptive statistics. Of the 294 articles initially identified, 18 were included in the review.

**Results:**

The majority of the studies (66.7%; *n* = 12) were qualitative. They focussed on five psychosocial domains – psychological including a lack of knowledge, misunderstanding and unmet information needs, the omnipresent experience of fear and sexual problems as well as social, cultural, spiritual and financial implications.

**Conclusion:**

Work focussing on the psychosocial implications of cervical cancer in women living in sub-Saharan Africa is limited. Only one study focussed specifically on a psychosocial domain, the rest reported little about psychosocial issues. There is an urgent need for research that focusses exclusively on psychosocial health, separate from other studies.

**Contribution:**

To the best of our knowledge, this is the first study synthesising research conducted on this specific topic. We mapped the extent of the current evidence base, identified gaps and highlighted areas requiring additional inquiry.

## Introduction

Recently, Uwayezu et al. (2022) made an urgent call for education and training in psychosocial care for nurses practising in cancer care settings in Africa. The basis for this call was the level of unmet supportive care needs and the high level of emotional distress patients living with cancer in sub-Saharan Africa (SAA) experience. This call is supported by Onyeka, Onu and Agom (2023), who in a scoping review focussing on the psychosocial needs of adult cancer patients living in SAA found ‘overwhelming’ evidence of high unmet psychosocial needs.

Africa, the world’s second-largest continent, is divided by the Saharan Desert into two culturally different geographical regions. The region north of the desert is culturally influenced by Arab culture and Islam, while the region south of the desert, sub-Saharan Africa (SSA) is the poorest region in the world (The New World Encyclopedia 2020). Living in SSA is not easy, especially for women who face unique challenges. For instance, women and girls form the majority of the agricultural workforce while being responsible for the health and welfare of their families. Also, 60% of the population living with HIV and AIDS in this region is women. The coronavirus disease 2019 (COVID-19) pandemic added to the dire situation of women by increasing teenage pregnancies and child marriages (Collins 2022). In addition, SSA has the highest cervical cancer incidence and mortality rates in the world, and the rates are higher in Eastern, Southern and Middle Africa (Sung et al. 2021). Where the incidence of cervical cancer has decreased in some developed regions, its incidence is increasing in certain regions of SSA (Stelzle et al. 2021).

Although Human Papillomavirus (HPV) is necessary for cervical cancer to develop, it is not sufficient to cause cervical cancer. Co-infections with sexually transmitted diseases, such as HIV and *Chlamydia trachomatis*, and other factors, such as the long-term use of oral contraceptives, a higher number of childbirths and smoking, also play a role (Sung et al. 2021). Women living with HIV are six times more likely to develop cervical cancer, as they are more likely to acquire HPV infections and less likely to spontaneously clear the infection (Stelzle et al. 2021). In addition, cervical cancer can occur up to 10 years earlier in women living with HIV compared to those who are HIV negative (Snyman 2013).

It is well known that cervical cancer can be prevented by means of the HPV vaccine and screening. However, Ngwa et al. (2022) when investigating the state of cancer in SAA found this region lacks a proactive approach to cancer, and primary and secondary prevention services are poorly developed. In addition, when investigating aspects of HPV vaccination in Africa, Zhuang, Goyal and Xu (2019) found only 1% to 2% of females between the ages of 10 and 20 get vaccinated for HPV, screening is primarily opportunistic, and only a small percentage of women, approximately 19%, have been screened (Sung et al. 2021). Cancer diagnosis and treatment also pose challenges as SAA has the lowest available facilities rendering these services of any region of the world. Many patients present with advanced cancer and do not complete their treatment, as they personally have to pay for the healthcare services – something they cannot afford (World Health Organization Regional Office for Africa 2022).

Psychosocial well-being requires a person to cope with the stresses of everyday living and realise his and her full potential as a productive member of society including the physical, economic, social, mental, emotional, cultural and spiritual determinants of health (Kumar 2020). A cancer diagnosis influences the psychosocial well-being of all people living with cancer and they experience high levels of psychosocial distress throughout the cancer journey (Gorman 2018). According to Holland (Gorman 2018), the type of cancer, personal coping skills and society’s attitudes towards the disease influence the extent to which a person would adapt to this distress. Maree, Holtslander and Maree (2021a), when synthesising the experiences of women living with cervical cancer in Africa, found that the psychosocial implications of cervical cancer begin before diagnosis, often at the first experience of symptoms and continue well after treatment is completed. However, little is known about what these implications entail.

To address this knowledge gap, we selected a scoping review method. This method was preferred to a systematic review as the context of our study was SSA, which did not allow us to retrieve international evidence. In addition, we did not wish to answer questions about nursing practice and conflicting results (Munn et al. 2018). Scoping reviews allow researchers to review the extent, range and nature of research activities and include both qualitative and quantitative work, regardless of its quality. Scoping reviews also identify gaps in the existing evidence base and highlight areas needing additional enquiry. It can also be helpful to practitioners who might not have the time or resources to read the available literature (Arksey & O’Malley 2005; Pollock et al. 2021). For the purpose of the study, we included the psychosocial constructs psychological, social, spiritual, cultural and financial underpinned by the studies of Uwayezu et al. (2022) and Kumar (2020) in our search.

### Review purpose and questions

The purpose of our scoping review was to describe the publication output reporting the psychosocial implications of cervical cancer in women living in SSA. To fulfil the purpose of the review, we posed the following research questions:

- What are the research outputs reporting psychosocial implications of cervical cancer for women living with cervical cancer in SSA between 01 January 2012 and 31 December 2021?
- What are the trends and knowledge gaps in this field of study in SSA in the last decade?
- What is the guide for future research into the psychosocial implications of cervical cancer in SSA?

## Research methods and design

We used the methodological framework of Arksey and O’Malley (2005), refined by Levac, Colquhoun and O’Brien 2010), to guide our review. This framework consists of five steps. In Step 1, there is the formulation of the research question(s). Step 2 consists of a broad search to identify the relevant studies. Step 3 consists of a selection of the studies based on the inclusion and exclusion criteria, while Step 4 consists of charting the data by means of a charting form. Step 5 collates, summarises and reports the results.

We used the keywords Africa and cervical cancer in combination with psychosocial, psychological, social, spiritual, cultural and financial to search the databases Cumulative Index to Nursing and Allied Health Literature (CINAHL), Pubmed, PsychINFO, Scopus and Web of Science. We also hand-searched two key journals and the reference lists of tracked articles to identify additional studies. Studies were included if published in peer-reviewed journals in English, focussed on women diagnosed with cervical cancer, included psychosocial aspects related to cervical cancer and published between 01 January 2012 and 31 December 2021. This 10-year period was chosen as a Pubmed search showed a dearth of research before 2012. As we aimed to include only peer-reviewed, published research, we excluded literature reviews, dissertations, editorials and letters to the editor, case reports, discussion papers, conference abstracts and grey literature.

**FIGURE 1:**
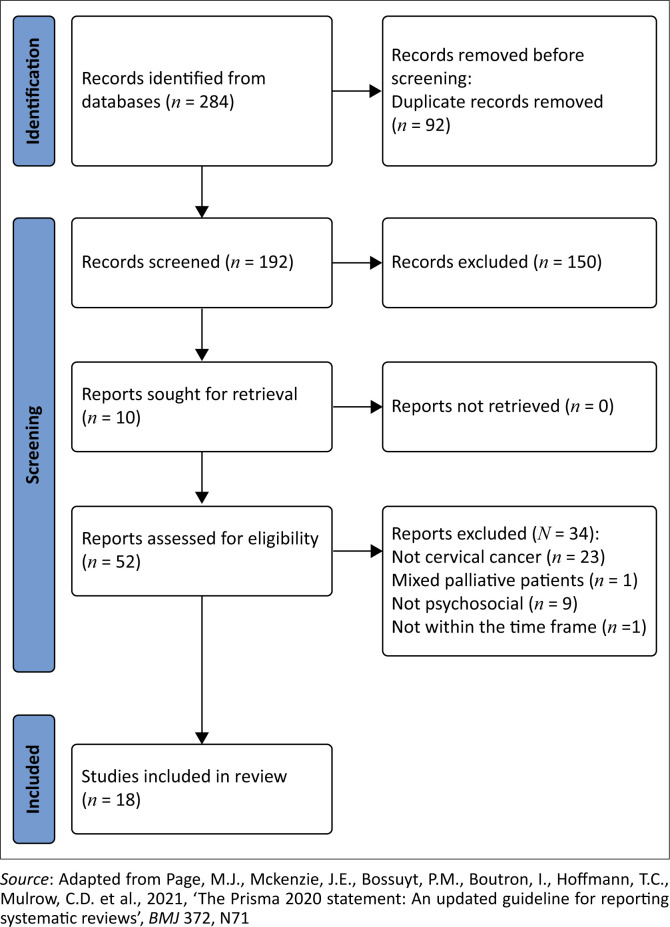
PRISMA flow diagram illustrating the review flow process.

Data collection began in July 2022 and ended in September 2022. This process included the importing of the lists of publications into an electronic reference manager and then onto an Excel spreadsheet. The database search produced 284 manuscripts (*n* = 284), the key journal search produced six (*n* = 6) articles and four were identified by means of the reference lists (*n* = 4), totalling 294 (*n* = 294). Ninety-two articles (*n* = 92) were duplicates and therefore removed. We excluded 150 articles (*n* = 150) as they did not meet the inclusion criteria, based on their titles or the abstracts if the titles were not conclusive, thus 52 (*n* = 52) remained. After screening the full texts of these articles, 34 articles were excluded and 18 (*n* = 18) were included in the review. Two researchers independently applied the selection criteria and a consensus meeting confirmed the work included in the review. A PRISMA flow diagram (Figure 1) presents the details of the search output.

To allow us to capture the data of the work included in the review, we developed a charting form and captured the authors, year of publication, country where the study was conducted, journal of publication, study purpose, study population, sampling method, sample size, data collection method and instrument, data analyses method and the major results (see Online Appendix 1). A separate data extraction sheet, supporting a template analyses style (Brooks et al. 2015), extracted the reported psychosocial issues. We used descriptive statistics and content analyses to analyse the data.

To enhance the rigour of the study, we measured our study against an evidence-based checklist, aimed at improving the quality of scoping reviews, published by Cooper et al. (2019). We also used an iterative team approach (Levac et al. 2010) by refining the previous step before commencing to the next step.

A.A.H. approved the final draft.

### Ethical considerations

This article followed all ethical standards for research without direct contact with human or animal subjects.

## Results

### Countries where the studies were conducted, years and journals of publication

The studies originated from six (*n* = 6) sub-Saharan countries; most were conducted in South Africa (50%; *n* = 9), Ghana published three studies (16.7%; *n* = 3), Ethiopia and Zambia two each (11.1%; *n* = 2) and Kenya and Rwanda one article (6.3%; *n* = 1). The highest percentage (22.2%; *n* = 4) was published in 2015, followed by 2018 and 2019 when there were three studies (16.7%; *n* = 3) published, respectively. Two studies (11.1%; *n* = 2) were published in 2016 and 2020, respectively, while the years 2012, 2013, 2014 and 2017 saw the publication of only one study (5.3%; *n* = 1). There was no publication found dated 2021.

The articles were published in 16 (*n* = 16) different journals, of which the majority (72.2%; *n* = 13) were published in international journals, while the rest (27.8%; *n* = 5) were published in journals based in SSA. Only two journals, both based in SSA, published more than one study – the International *Journal of Africa Nursing Sciences* published two (11.1%; *n* = 2), and another two (11.1%; *n* = 2) appeared in the *Southern African Journal of Gynaecological Oncology*. Only one study (5.6%; *n* = 1) was published in a journal specifically dedicated to psychology or psychosocial topics, four (22.2%; *n* = 4) in journals focussing on cancer and three articles (16.7%; *n* = 3) appeared in palliative and supportive care journals.

### Themes investigated

There was a wide variety of themes identified. Experiences were the theme most commonly identified and investigated in six (33.3%; *n* = 6) studies. Barriers or challenges were the focus of five studies (27.8%; *n* = 5), while three studies (16.7%; *n* = 3) investigated quality of life. Table 1 provides the details.

**TABLE 1:** Themes and subthemes investigated (*N* = 18).

Variables	*n*	%
**Experiences**		
Cervical cancer in general	2	11.1
Communication	1	5.6
High-dose rate brachytherapy	1	5.6
Life partner support	1	5.6
Late side effects of treatment	1	5.6
**Barriers or challenges**		
Screening, diagnosis and treatment	2	11.1
Financial or socio-economic	2	11.1
Delayed consultation	1	5.6
Coping strategies	1	5.6
Cultural factors	1	5.6
Informational needs	1	5.6
Knowledge	2	11.1
Sexual functioning	1	5.6
Spiritual meanings	1	5.6
Quality of life	3	16.7

### General characteristics of the work

The majority of the studies (66.7%; *n* = 12) were qualitative, five (27.8%; *n* = 5) were quantitative, while one (5.6%; *n* = 1) used a mixed methods design. All the studies included women diagnosed with cervical cancer (100%; *n* = 18); however, three studies (16.7%; *n* = 3) included other participants, namely caregivers, key informants and unscreened women. The populations for the studies consisted of women receiving treatment for cervical cancer (33.3%; *n* = 6), women attending gynaecological and/or radiotherapy units (22.2%; *n* = 4), women undergoing follow-up treatment (11.1%; *n* = 2) and survivors (11.1%; *n* = 2), as well as women visiting the hospital, women with an early stage of cervical cancer, newly diagnosed patients, cervical cancer patients attending a hospice, patients admitted to an inpatient unit and women referred for palliative care.

All the studies used non-random sampling. Sampling was primarily purposive, and 11 studies (61.1%; *n* = 11) used this sampling method, five studies used convenience sampling (27.8%; *n* = 5), two studies consecutive sampling (11.1%; *n* = 2) and the (the sampling method) of one study (5.6%; *n* = 1) was not clear. The sample sizes of the qualitative samples (*n* = 15) ranged from 4 to 40, average 17.3 (s.d. ± 8.1). Two hundred and sixty women (*n* = 260) took part in the qualitative work. The sample sizes of the quantitative studies (*n* = 6) ranged from 147 to 404, an average of 243 (s.d. ± 103.8) and there were 1458 (*n* = 1458) respondents included in the quantitative work.

Interviews were the most common method of data collection and were used in more than 80% (81.3%; *n* = 13) of the studies. Similarly, interview guides or schedules were the most popular data collection instruments (44.4%; *n* = 8), and thematic analyses were the most commonly used data analysis method (55.6%; *n* = 10). Table 2 presents the details.

**TABLE 2:** Data collection and analysis methods used (*N* = 18).

Variables	*n*	%
**Data collection methods**		
Interviews		
In-depth	5	27.8
Structured	2	11.1
Semi-structured	2	11.1
Unstructured	2	11.1
Face-to-face	1	5.6
Qualitative	1	5.6
Individual and group	1	5.6
Focus group discussions	2	11.1
**Data collection instruments**		
Questionnaires		
Non-specific	1	5.6
Researcher administered	3	16.3
Self-administered	1	5.6
Focus group guide	1	5.6
Interview guides or schedules	8	44.4
One opening question	3	16.7
Theme lists	1	5.6
Not clear	1	5.6
**Data analyses methods**		
Descriptive statistics	4	22.2
Descriptive and inferential statistics	1	5.6
Inductive approach	1	5.6
Thematic analyses	10	55.6
Open coding and template analyses	2	11.1
Not clear	1	5.6

### Psychosocial implications

Seven themes describing the psychosocial implications of cervical cancer were identified. This is summarised in Box 1.

BOX 1 Themes illustrating the psychosocial implications identified.

Lack of knowledge, misunderstanding and unmet information needs

The omnipresent experience of fear

Sexual problems

The social implications of cervical cancer

The cultural implications of cervical cancer

The spiritual implications of cervical cancer

The financial implications of cervical cancer

#### Lack of knowledge, misunderstanding and unmet information needs

Half of the studies (50%; *n* = 9) reported women’s lack of knowledge. The identification of knowledge deficits was in terms of the prevention of cervical cancer, its causes, risk factors, screening and treatment options (Benemariya et al. 2018; Binka, Doku & Awusabo-Asare 2017; Binka et al. 2018, 2019; Maree & Kaila 2014; Maree, Langley & Nqubezelo 2015; Tadesse 2015), brachytherapy (Dzaka & Maree 2016) and the late effects of the disease and treatment (Ntinga & Maree 2015). A lack of knowledge resulted in delayed health-seeking (Benemariya et al. 2018; Binka et al. 2019), mistrust and fear (Binka et al. 2018; Maree & Kaila 2014) and finally misunderstanding (Maree et al. 2015).

Numerous studies described the unmet information needs about treatment and the late effects of treatment (Dzaka & Maree 2016; Long, Freidrich-Nel & Joubert 2016; Maree & Kaila 2014). Caren, Mose and Kurgat (2020), when investigating communication among patients and their caregivers, described collusion and the withholding of information about the disease and treatment, even deliberately, that resulted in loneliness, bitterness and feelings of being cheated and betrayed. It was also found that a lack of information hampered the relationship between patients and their caregivers.

#### The omnipresent experience of fear

The majority of the studies (66.7%; *n* = 12) described fear as one of the psychosocial implications of cervical cancer. Araya et al. (2020), in their study focussing on health-related quality of life (HRQoL) among cervical cancer patients in Ethiopia, found 60.4% of the 404 women (*n* = 404) included in their study experienced slight to severe problems with anxiety and/or depression. Fear seemed an ever-present feeling, which began when the women noticed the signs of cervical cancer (Binka et al. 2018). Fear also hindered some of the women from seeking cancer care, as they were scared others, even their husbands, would find out they had cancer, would reject them and they would become outcasts in the community. They also did not trust healthcare providers and some did not consult at nearby healthcare facilities because of their fear of confidentially not being maintained (Benemariya et al. 2018; Caren et al. 2020).

Fear also presented itself in the form of mistrust, as the women feared the possible attitudes of healthcare workers and misdiagnosis, resulting in unnecessary further consultations and tests (Binka et al. 2019). The women experienced fear when the news was broken to them (Maree et al. 2015), fearing the future, the unknown and death (Caren et al. 2020; Maree & Kaila 2014), the treatment and side effects (Binka et al. 2017, 2018) and some feared Western medicine would not work and reverted to herbal medicine including locally made medicine from barks, leaves and roots or trees (Binka et al. 2018).

Both surgery (Caren et al. 2020) and radiotherapy created fear (Maree, Mosalo & Wright 2013), and the women were scared when they arrived at the radiotherapy department (Long et al. 2016). Some women were afraid of brachytherapy, before and after receiving the first treatment (Dzaka & Maree 2016). The possibility of recurrence also created fear and anxiety; some women feared the disease would return when they resumed sexual activities (Tadesse 2015), while others wished for reassurance that the cancer would not return (Maree et al. 2015). These findings are supported by Tadesse (2015), who found women experience constant anxiety because of the fear of recurrence.

#### Sexual problems

Sexual problems commenced when the symptoms of cervical cancer were experienced (Tadesse 2015) and were reported in 55.6% (*n* = 10) of the studies. Fakunle and Maree (2019), in a study focussing on the sexual functioning of women receiving treatment for cervical cancer and beyond, found that 94.6% of the sample experienced sexual dysfunction, which persisted over time. In addition, Sabulei and Maree (2019) found all the domains of sexual functioning, sexual activity and enjoyment and vaginal functioning declined after treatment. In addition, du Toit and Kidd (2015) found HIV-positive women experienced no improvement in any of the sexual domains over the study period, extending from diagnosis to 3 months follow-up. Araya et al. (2020) and Binka et al. (2017) supported these findings and confirmed women experienced challenges with engaging in sexual activity because of the side effects of the disease and treatment; some even abstained because of the fear of bleeding (Binka et al. 2018). However, sexual dysfunction was not without implications and was of great concern to women as they risked losing their partners (Ntinga & Maree 2015; Maree et al. 2013) some even ended their marriages because of unwillingness to continue with sexual intercourse after being diagnosed with cervical cancer (Tadesse 2015).

#### The social implications of cervical cancer

The social implications of cervical cancer featured in seven (43.8%; *n* = 7) studies. Many of the women faced the risk of rejection and social isolation. The negative societal attitude, stigma and lack of knowledge influenced the women in various ways. Some isolated themselves because of the smell of the vaginal discharge (Maree & Kaila 2014; Tadesse 2015) while others wanted to hide their diagnosis from their extended family (Tadesse 2015). Some lacked support from their families (Binka et al. 2017) and some lost their friends, as the friends were scared they could catch the disease. The women also lost their social lives and social support. Misinformation was rife, and the misinformation shared by community members resulted in hesitancy to be treated and even suicidal thoughts; the women were even told they were going to die (Maree & Kaila 2014). In addition, those who completed treatment and consulted at primary health clinics were labelled as cancer patients and referred to the hospital for treatment of minor ailments. Sabulei and Maree (2019), when exploring the quality of life of women diagnosed with cervical cancer, found social functioning scored the lowest of all QoL domains and did not improve during the 12 months after treatment. Du Toit and Kidd (2015) found a similar trend in HIV-positive women, and Araya et al. (2020), in a similar study, found a low social functioning score.

#### The cultural implications of cervical cancer

Three studies (16.7%; *n* = 3) described cultural issues specifically. Tadesse’s study (2015) conducted in Ethiopia, highlighted the early age of marriage, as early as 8 years, on average 16 years and the sexual exposure of a polygamous marriage as cultural vulnerabilities. Being married at such a young age compromised girls’ chances of education, exposure to information and the ability to care for themselves economically and health-wise. In addition, men were the decision makers and some women had to ask permission to consult for healthcare. As married men were responsible for the cost of healthcare for their wives and children, they delayed or failed to support their wives financially who in turn delayed seeking healthcare (Binka et al. 2019).

Traditional healers and medicine also played a role. Caren et al. (2020) found some cultural groups believed hospitals cannot treat cancer and that one is merely digging one’s own grave by having hospital treatment. Traditional healers seemed to be the first port of call (Maree et al. 2013) and women visited traditional healers until cervical cancer-related complications arose (Benemariya et al. 2018; Binka et al. 2019). Some women believed traditional medicine was more potent than Western medicine and consulted traditional healers when mismanaged at healthcare facilities (Binka et al. 2018, 2019). Some women believed witchcraft was responsible for their cervical cancer in an attempt to prevent blame, rejection and isolation (Mabena & Moodley 2012). Women, especially older women, were ashamed and having to consult with doctors when experiencing problems involving private parts hindered seeking healthcare. Some women had to ask permission to consult, as men may not touch a woman’s private parts without her husband’s permission. In addition, the need to avoid being seen by younger people and the association of cervical cancer with promiscuity further hindered individuals from seeking healthcare (Binka et al. 2019).

#### The spiritual implications of cervical cancer

Only one of the studies (5.6%; *n* = 1) (Mabena & Moodley 2012) investigated spirituality, while some of the qualitative work described some spiritual implications of cervical cancer. Binka et al. (2019) found women believed God was punishing them for wrongdoing and blamed themselves for having cervical cancer. These women managed their disease by means of divine intervention rather than going to the hospital for treatment. Mabena and Moodley (2012) found that because of the overwhelming nature of a cervical cancer diagnosis, women initially sought spiritual meanings for their illness and considered it as God’s will, God’s way of testing them. Dzaka and Maree (2016) found women receiving brachytherapy described it as Hell and believed only God could help them to get through the treatment. Ntinga and Maree (2015), when describing the late effects of cervical cancer and its treatment, found the women asked ‘Why?’, which could be a sign of spiritual distress.

#### The financial implications of cervical cancer

The financial hardship of having cervical cancer was described in the majority (77.8%; *n* = 14) of the studies. Araya et al. (2020) found financial concerns the most troublesome of all the HRQoL domains. Sabulei and Maree (2019) found a similar trend, especially during the time of treatment, while Benemariya et al. (2018) found finances were the main reason for delayed healthcare seeking. Women were not able to work during the time of treatment and even afterwards and lost their income (Binka et al. 2017, 2018; Ntinga & Maree 2015; Tadesse 2015). Some had no formal employment or steady income and could not afford treatment (Caren et al. 2020; Tadesse 2015), while others had to abandon treatment and were even abandoned while in hospital for treatment because of the cost (Owenga & Nyambedha 2018). Some women receiving treatment could not afford the transport costs involved (Dzaka & Maree 2016; Maree et al. 2013), while those who completed treatment missed follow-up care because of the same reason (Ntinga & Maree 2015). The financial implications of cervical cancer forced some women to forfeit the healthcare they preferred (Maree et al. 2015), sell their property and have family members begging in the streets to be able to pay for treatment (Tadesse 2015).

The women depended on their husbands for financial support (Tadesse 2015) and also for paying for screening and treatment (Binka et al. 2019), which led to constant clashes with husbands because of the cost sustained since diagnosis (Tadesse 2015). Not all husbands were willing to support their wives financially, and some women had to ask other family members, the church and their children for assistance, which was not sufficient to meet their needs (Maree et al. 2013; Owenga & Nyambedha 2018). Affording basic commodities, including sanitary protection and personal hygiene items, also poses a problem (Binka et al. 2018; Ntinga & Maree 2015; Owenga & Nyambedha 2018).

## Discussion

Only a small number of studies, 18 in total, published over a 10-year period, could be found. In addition, only one of the studies focussed on a psychosocial domain specifically, while information about psychosocial issues was reported as part of other investigations. Screening for cervical cancer and living with a cervical cancer diagnosis were also investigated in the same study, which complicated the abstraction of the psychosocial issues women living with cervical cancer experienced.

The research output, originating from South Africa, Ghana, Ethiopia, Zambia, Kenya and Rwanda, did not represent the incidence of cervical cancer in SSA. Eswatini has the highest incidence of cervical cancer in the world followed by Malawi, which also has the highest mortality rate, which is seven times the global rate (Gerstl et al. 2022). Eswatini supports citizens to access specialist healthcare, such as cancer care in neighbouring countries, while Malawian citizens do not have access to radiotherapy, thus hampering curative treatment (Masamba 2015). Having to travel to other countries for treatment and/or having the prospect of not receiving standard treatment could have devastating implications on psychosocial health; however, there was no investigation into this. In addition, recent reports indicated an increase in the incidence of cervical cancer in seven sub-Saharan countries – Gambia, Kenya, Malawi, Seychelles, South Africa, Uganda and Zimbabwe (Sung et al. 2021), while only two of these countries reported issues pertaining to the psychosocial implications of a cervical cancer diagnosis. Unfortunately, research funding is the major barrier hampering cancer research output in Africa, as the little research funding available is primarily provided by governments and international funders (Kayamba et al. 2021), which could explain the low and skewed research output.

Considering the nature of the psychosocial domain, it seems logical that most of the studies were qualitative, as qualitative work attempts to understand people’s experiences, beliefs, behaviours, attitudes and interactions (Gray, Grove & Sutherland 2021). However, the quantitative work added important information about the quality of life and sexuality, how the domains and symptoms differ between women diagnosed with HIV and those who are HIV negative, and how the domains changed during and after having had treatment. As supported by other reviews conducted in Africa (Maree et al. 2021a; Maree, Khutjwe & Swart 2021b; Maree & Schmollgruber 2014), interviews were the most popular data collection method, which was also used in some of the quantitative work. As the average literacy level of sub-Saharan African women 15 years and older is 59% (The World Bank 2022), interviews are an appropriate data collection method as they do not consider literacy and/or mother tongue (Gray et al. 2021).

Our study provides evidence that the lack of knowledge of cervical cancer remains a problem, influencing health-seeking behaviour and resulting in mistrust and fear. However, despite being widely publicised and well known, investigations continue into the lack of knowledge of cervical cancer. What is concerning is the finding that patients are not informed about their diagnosis and are not provided with the information they need to have control over their own lives. Truth telling, originating from the ethical norm autonomy (Varkey 2021), obliges healthcare practitioners to provide patients with the information they want or need in a way they understand (Athanas, Gasto & Renatha 2020). However, in clinical practice, there is often conflict between the ethical principles of beneficence and autonomy (Varkey 2021), complicating truth telling and information giving, as healthcare practitioners want to protect patients from difficult unpleasant emotions and foster hope (Sarafis et al. 2013). Even though sharing information about a cancer diagnosis is influenced by a cultural communication style (Athanas et al. 2020), cancer patients across cultures want more information about their diagnosis and prognosis (Sarafis et al. 2013) and as seen from our study, withholding information caused unnecessary emotional pain.

The work included in this review highlighted the fear of cervical cancer treatment, especially brachytherapy; however, this fear subsided while the fear of recurrence, also influenced by culture (Anderson et al. 2021), continued and created constant anxiety. This finding contrasts with that of a systematic review on the fear of recurrence, including work from the developed world only (Simard et al. 2013). This study reported a low to moderate level of fear of recurrence; however, this fear was one of the top concerns identified and the most frequently unmet need found in the current study. In addition, the fear of recurrence remained stable and did not change during survivorship. Whether this trend would apply to people living with cancer in Africa, specifically those diagnosed with cervical cancer, remains unknown, as there is no research answering this question available.

As seen in our study, experiencing the symptoms of cervical cancer and having had treatment for cervical cancer lead to sexual problems. Having sexual problems threatened women’s relationships with their husbands or partners, and they feared their spouses would reject and abandon them, which did happen for some. There is much description of sexual problems because of the treatment of cervical cancer (Shankar et al. 2017), but, as supported by this study, many women depend on their life partners financially and losing their partners means losing their livelihoods. In addition, as seen from our study, being financially dependent hampered the women’s health-seeking behaviour and access to treatment. Denny, Quinn and Sankaranarayanan (2006) support this finding and describe women living in developing countries as poor, uneducated and disempowered. Dominic, Ogundipe and Ogundipe (2019) found having a bank account, being part of the labour force, having basic education and being the head of a household enhanced women’s decisions to access healthcare facilities. This is a complex issue involving cultural beliefs but could add to the body of knowledge if there could be comparisons in terms of cervical cancer.

Using traditional healers and medicines as the first port of call when experiencing the signs of cervical cancer delayed diagnosis and the treatment available. However, this is nothing new, as according to Matsheta and Mulaudzi (2008), black women consult traditional healers before they consult healthcare professionals. The authors also found South African traditional healers have good knowledge of cervical cancer care. This is in contrast with the Ugandan study of Mwaka et al. (2021) who found traditional healers considered cancer as a new and challenging disease and perceived gynaecological cancers, especially as sexually transmitted or the result of dirt accumulation. To discuss the value of traditional healing and medicines in healthcare is beyond the scope of this study; however, of note is that women used traditional healers and medicines when the healthcare system failed them (Van Schalkwyk, Maree & Dreyer Wright 2008), which could result in negative disease outcomes.

As evidenced by our study, some women blamed themselves for their cervical cancer diagnosis, as they believed God was punishing them for wrongdoing. Anorlu (2008), in a sub-Saharan perspective of cervical cancer, supports this finding as does Shinan-Altman, Levkovich and Hamama-Raz (2022) in a study conducted in Israel. However, the self-blame was because of ignoring the symptoms of cervical cancer and not because of God’s wrath. Block, Dafter and Greenwald (2006) warn that self-blame can persist and lead to helplessness, which are issues needing investigation before conclusions can be made.

### Strengths and limitations

We used a traditional scoping review method and included only peer-reviewed articles. The use of specific keywords and selected databases, along with reference list searches and the selection of key journals based in Africa, may have resulted in some relevant work being overlooked. Additionally, the reporting of psychosocial issues as part of other investigations and the mixing of studies involving women with and without cervical cancer complicated data extraction, potentially causing us to miss important implications. However, to the best of our knowledge, this is the first study synthesising research on the psychosocial implications of cervical cancer for women living in SAA.

## Conclusion

The study provided evidence that work focussing on the psychosocial implications of a cervical cancer diagnosis in women living in SSA is limited, as only 18 articles could be included in this review. The work did not represent the countries with the highest incidence and mortality rates or those without standard treatment that must refer women to other countries for care. Only one study focussed specifically on a psychosocial domain; the rest investigated other topics, such as screening, in addition to a cervical cancer diagnosis and tended to report little about psychosocial issues per se. The quantitative studies primarily examined quality of life. Interviews were the most commonly used data collection method, with only non-random sampling employed. Fifty per cent of the studies reported a lack of knowledge about cervical cancer; however, none included a program to improve knowledge and health-seeking behaviour, which is crucial for addressing psychosocial outcomes for patients. There is an urgent need for such initiatives. The psychosocial implications for women who were unable to receive standard treatment because of unavailability or cost were not investigated and need prioritisation. Similarly, studies on communication, information sharing and truth telling are needed to improve clinical practices and the psychosocial health of those affected. As the fear of recurrence creates anxiety and depression that do not subside over time, longitudinal work is necessary to explore this phenomenon and determine how to support patients on a long-term basis. Lastly, we urgently need research that focusses on the psychosocial health, implications and support needs of these women, which are not embedded in other studies.
